# Association between Obstructive Sleep Apnea and Type 2 Diabetes Mellitus: A Dose-Response Meta-Analysis

**DOI:** 10.1155/2021/1337118

**Published:** 2021-09-30

**Authors:** Zhixiang Yu, Jin-Xiang Cheng, Dong Zhang, Fu Yi, Qiuhe Ji

**Affiliations:** ^1^Nephrology Department, Xijing Hospital, The Air Force Military Medical University, Xi'an 710032, Shaanxi Province, China; ^2^Department of Neurology, Tangdu Hospital, The Air Force Military Medical University, Xi'an 710038, Shaanxi Province, China; ^3^Department of Cardiology, Xijing Hospital, The Air Force Military Medical University, Xi'an 710032, Shaanxi Province, China; ^4^Department of Endocrinology and Metabolism, Xijing Hospital, The Air Force Military Medical University, Xi'an 710032, Shaanxi Province, China

## Abstract

**Materials and Methods:**

We screened four databases (PubMed, Embase, Cochran Library, and CNKI) for the observational studies about the OSA and T2DM. Studies were collected from database establishment to October 2020. We performed a traditional subgroup meta-analysis. What is more, linear and spline dose-response models were applied to assess the association between apnea-hypopnea index (AHI), an indicator to evaluate the severity of OSA, and the risk of T2DM. Review Manager, version 5.3, software and Stata 16.0 were used for the analysis.

**Result:**

Seven observational studies were included in the research. We excluded a study in the conventional meta-analysis. In the subgroup analysis, mild-dose AHI increased the risk of T2DM (odds ratio = 1.23, 95% confidence interval = 1.06–1.41, *P* < 0.05). Moderate-dose AHI increased the risk of T2DM with a higher odds ratio (OR = 1.35, 95% CI = 1.13–1.61, *P* < 0.05). Moderate-to-severe-dose AHI increased the risk of T2DM with a higher odds ratio (OR = 2.14, 95% CI = 1.72–2.67, *P* < 0.05). Severe-dose AHI increased the risk of T2DM with a higher odds ratio (OR = 2.19 95% CI = 1.30–3.68, *P* < 0.05). Furthermore, the spline and linear dose-response meta-analysis results revealed that the risk of T2DM increased with increasing AHI values.

**Conclusion:**

Through the dose-response meta-analysis, we found a potential dose-response relationship existed between the severity of OSA and the risk of T2DM. This relationship in our passage should be considered in the prevention of T2DM in the future.

## 1. Introduction

Obstructive sleep apnea (OSA) is one of the sleep disorders due to the refractory hypoxemia episodes and sleep fragments, leading to daytime sleepiness, impaired performance, and reduced quality of life.

There are 90% elderly males and 78% elderly females suffering from OSA approximately worldwide [1]. The apnea-hypopnea index (AHI) is an indicator of OSA's severity and whether or not to treat OSA. Mild OSA patients with AHI (5–15) without a comorbidity do not need to treat with continuous positive airway pressure (CPAP). Moderate OSA patients with AHI (15–30) without a symptom need to treat with CPAP [2]. Respiratory disturbance index (RDI), the sum of the total number of respiratory disturbances per hour, is another indicator for evaluating OSA's severity. In this research, we adopted the formula *b*=*rS*_*y*_/*S*_*x*_ to estimate the corresponding AHI [3]. Intermittent hypoxemia activates the sympathetic nervous system. It increases catecholamine levels, decreasing insulin sensitivity and promoting pancreatic beta-cell apoptosis [4], suggesting a possible mechanism underlying OSA's association with T2DM.

Type 2 diabetes mellitus (T2DM) is a systemic disease and a massive health threat, causing considerable damage to each organ. The incidence of T2DM is still rising, which has become severe and expanding worldwide wellbeing burden. The global figures read that 381.8 million adults are affected, and these data will come to 591.9 million in 2035. In developed countries, most T2DM patients are older than 50, while in developing countries, 41% of T2DM are elderly [5]. Previous studies found that T2DM and OSA shared the same high-risk group [1].

According to previous research works, OSA was closely associated with the high death rate from all causes. OSA damages all the systems in human bodies and organs with significant vascular structure, including the brain, heart, and kidneys. Besides, Xu et al. [6] reported that OSA was associated with metabolic syndrome, which revealed the potential relationship between OSA and endocrinology. In this research, we paid attention to the association between OSA and T2DM. Epidemiological research studies revealed that approximately 24%–86% of T2DM patients have OSA [7, 8].

OSA has been defined as a risk factor for T2DM by many studies [9]. Moreover, Abud et al. [10] reported that CPAP treatment would benefit T2DM patients with OSA. An OSA previous meta-analysis documented comorbidity of OSA and T2DM [11]. However, it is unknown whether the relationship between OSA severity and the risk of T2DM can be described with a linear or spline model.

In our present research, there were seven observational studies included. We planned to explore the association between OSA and different T2DM severity from these six OSA-related studies. To ensure our results' preciseness, we not only did a traditional meta-analysis but a dose-response meta-analysis was also accomplished. This study would offer strong evidence and new thought for the prevention of T2DM for OSA patients.

## 2. Methods

We registered this systematic review and dose-response meta-analysis with the INPLASY register (INPLASY2020110027). In addition, we followed the Preferred Reporting Items for Systematic Reviews and Meta-Analysis (PRISMA) guidelines for meta-analyses.

### 2.1. Study Selection

In the present study, we included cross-sectional studies, cohort studies, and case-control studies that had clear outcomes and reported hazard ratio (HR), odds ratio (OR), or relative risk (RR) and 95% confidence interval (CI) for the association between OSA and T2DM. HR and OR were considered approximately RRs [12]. The researchers used the AHI or RDI as the indicators were included. The studies included were all designed to be divided into subgroups by the AHI or RDI, and the mean AHI or RDI dose of each subgroup were clearly declared. There were no restrictions on gender.

Furthermore, the participants' ages were older than 18 years. We excluded patients with type 1 DM, and researchers used other indexes as indicators. As for the research type, case reports and reviews were also excluded. The criteria for inclusion and exclusion are shown in Table 1.

### 2.2. Search Strategy

We screened the PubMed, Embase, Cochrane Library, and CNKI (Chinese) databases. The retrieval time was from incipiency to September 2020. To fit different demands in different databases, we modified the search terms and strategy. In addition, we screened all references of the included articles to ensure we collected the related studies as many as possible. Moreover, we communicated with senior specialists when conceivable to complete the search methodology. The search strategy in PubMed is shown in Supplementary Materials 2.

### 2.3. Study Validation and Data Extraction

Two independent investigators (YZX and JXC) extracted data from the included articles. Discrepancies were handled by consultation and guide from JQH. In addition, data about baseline information of participants, study design, and relevant statistics were extracted. No qualification was made to measure the seriousness stratification of T2DM.

We evaluated the included studies according to the Newcastle-Ottawa scale (NOS) [13]. A quantitative scoring device proposed by the Cochrane Collaboration was adopted to assess the studies' methodological quality. The NOS contains three significant spaces: selecting subjects, comparability between bunches, and outcome measures. The most extreme of each region is four, two, and three. Thus, the lower the full score of the three parts, the worse the article is in methodological quality.

### 2.4. Data Synthesis and Analysis

Two investigators (YZX and YF) finished the conventional meta-analyses with Cochrane Review Manager, version 5.3, software to assess a specific outcome's risk.

The evaluation of heterogeneity among studies was carried out with the use of *Q* and *I*^2^. We adopt a standard for *P*-value that *P* value < 0.1 means the results possessed statistical heterogeneity. *I*^2^ describes the extent of variation due to heterogeneity rather than chance. The lower the *I*^2^ is, the less the variation is. *I*^2^ < 25% was considered little heterogeneity; 25% < *I*^2^ < 50%, a little heterogeneity. *I*^2^ > 50% showed there existed enough heterogeneity to select a random-effects model. While *I*^2^ < 50%, a fixed-effect model was employed [14].

Funnel plots were selected to assess whether the report existed publication bias. Egger's and Begg's texts were designed to recognize the plots' asymmetry, suggestive of bias. In this plot, *P* < 0.05 means the existence of a significant difference.

For further research, we performed a dose-response meta-analysis using Stata, version 16.0, software. We performed the dose-response meta-analysis based on a two-step method [15, 16]. First, the correlation between the AHI and the risk of T2DM was evaluated with a spline model [16]. In this spline model, we took AHI as an independent variable and RR as a dependent variable. Next, we selected a corresponding merge model to merge the risk value for each study calculated in the first step due to the heterogeneity. *α* = 0.05 was taken as a cut-off for the regression parameters [15]. For a single standard, RDI was translated to AHI as the equation *b* = *r*×*s*_*y*_/*s*_*x*_ [17]. According to Orsini et al. [12], the difference between HR, OR, and RR could be ignored in the dose-response meta-analysis. In our research, we considered HR, OR, and RR approximately the same.

### 2.5. Ethical Approval

This study complied with the Declaration of Helsinki. Given the study was a meta-analysis, no prior ethical approval was required.

## 3. Result

### 3.1. Literature Search

After a primary search, we identified 537 articles. There were 49 duplicated publications. In the left passages, from the title, we knew that there were 160 reviews. After abstracts screening, we excluded 290 studies, for they were not related to the topic. Then, we screened the whole text. We found 15 passages that did not mention the indicators we were interested in, and ten articles did not declare accurate RR/OR or 95% CI. Five research works did not perform OSA subgrouping. Natalia et al. [18] was a letter. No baseline data or characteristics were reported, so we excluded it. After screening the reference list for this research's integrality, we selected a passage for a supplement. Finally, seven articles were identified with our meta-analysis criteria in total (Supplementary Materials 1). The relevant ethics committee has approved all the studies included.

### 3.2. Characteristics and Quality of the Included Studies

All the research studies included in this meta-analysis were cohort studies, including 15252 participants. The study sizes were different from each other (303 to 8678). There existed a sex difference between the included research studies (women count 0 to 54.2%). As for the outcomes of the research works, 2381 new cases of T2DM were diagnosed (Table 2). The prevalence of T2DM in patients with OSA was approximately 15.61% (4.18% to 40.21%). We applied NOS to scale the included studies in Table 3. We evaluated the articles in three dimensions: selection quality, comparability, and outcome/exposure quality according to the corresponding criteria. Every asterisk represents one point, and we calculated the total points as the results of NOS for each study. The mean scores were 8 (7 to 9), which revealed that all the studies included were equipped with the wealthy quality for mixed analysis.

### 3.3. Meta-Analysis Results

**Figure 1:**
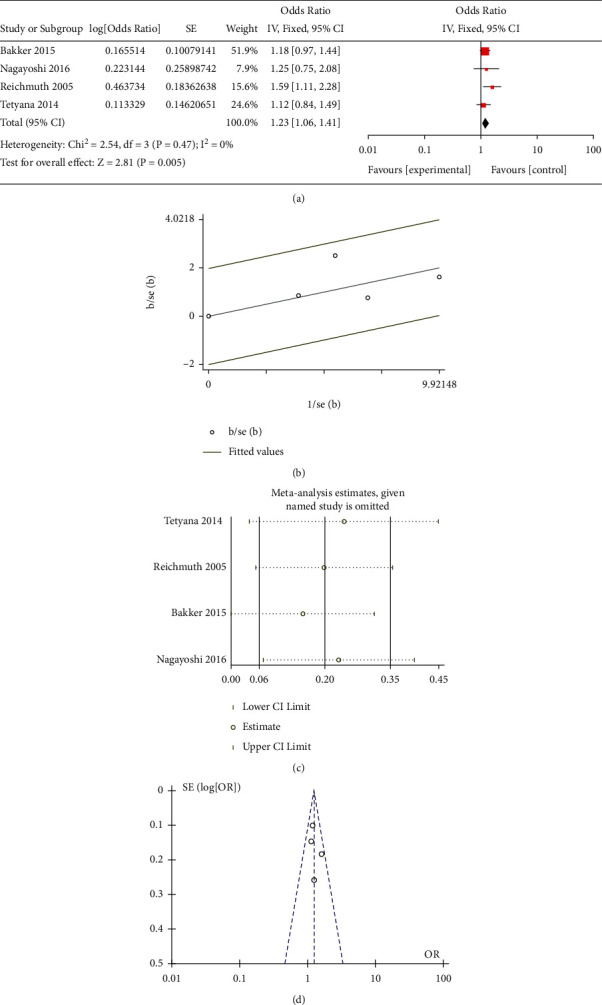
(a) Meta-analysis of mild-dose AHI and risk of T2DM using fixed-effects models. (b) Galbraith radial plot for assessment of publication bias among all included studies in the mild-dose subgroup meta-analysis. (c) Sensibility assessment of each included in the mild-dose subgroup meta-analysis. (d) Funnel plots for assessment of publication bias among all included studies in the mild-dose subgroup meta-analysis.

**Figure 2:**
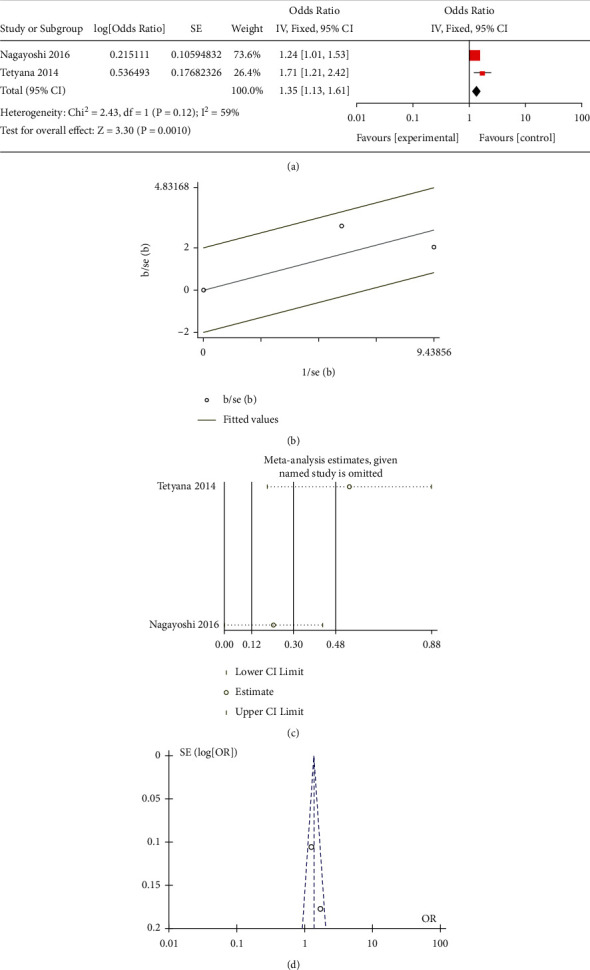
(a) Meta-analysis of moderate-dose AHI and risk of T2DM using fixed-effects models. (b) Galbraith radial plot for assessment of publication bias among all included studies in the moderate-dose subgroup meta-analysis. (c) Sensibility assessment of each included in the moderate-dose subgroup meta-analysis. (d) Funnel plots for assessment of publication bias among all included studies in the moderate-dose subgroup meta-analysis.

**Figure 3:**
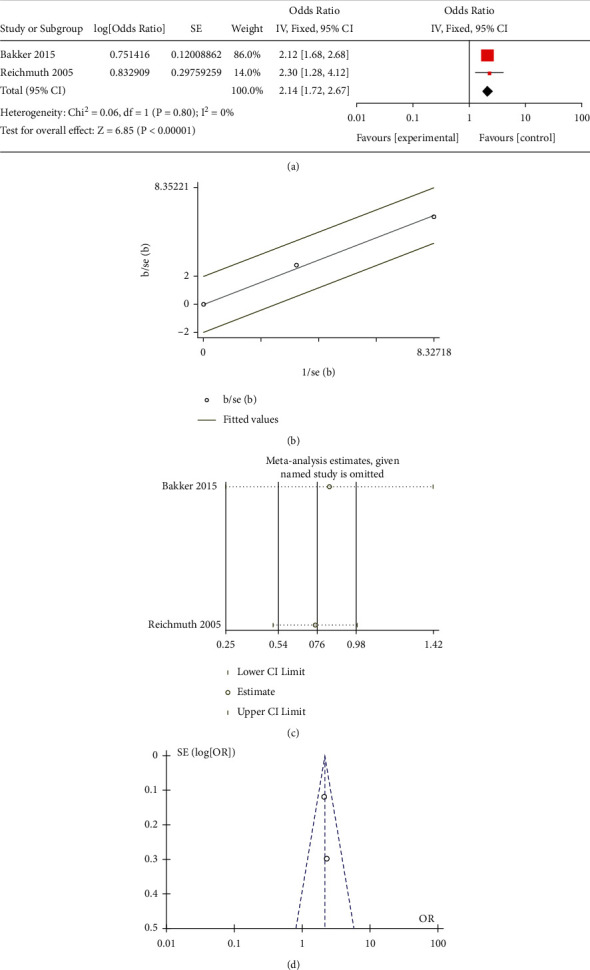
(a) Meta-analysis of moderate-to-severe-dose AHI and risk of T2DM using fixed-effects models. (b) Galbraith radial plot for assessment of publication bias among all included studies in the moderate-to-severe-dose subgroup meta-analysis. (c) Sensibility assessment of each included in the moderate-to-severe-dose subgroup meta-analysis. (d) Funnel plots for assessment of publication bias among all included studies in the moderate-to-severe-dose subgroup meta-analysis.

**Figure 4:**
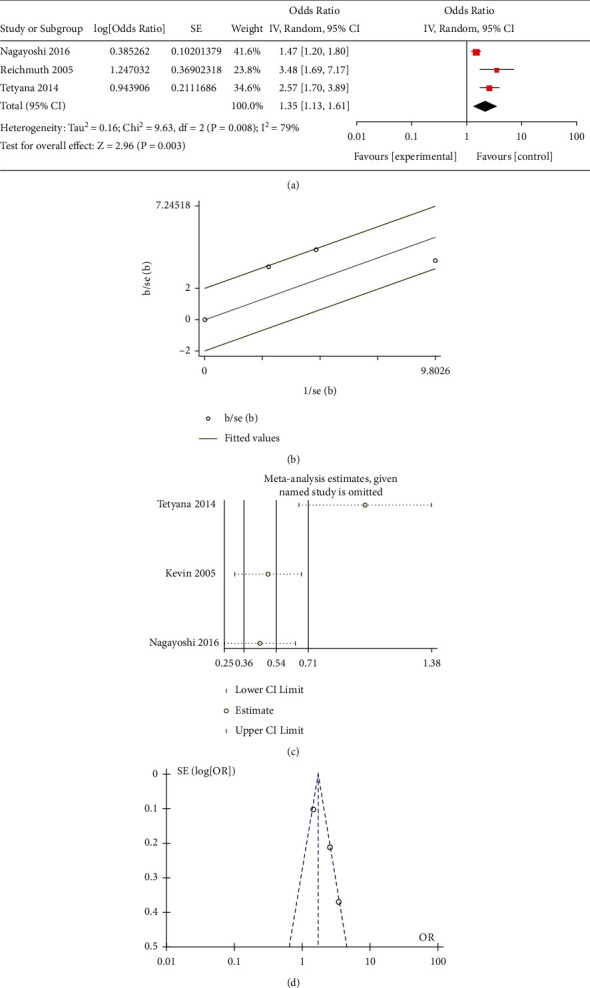
(a) Meta-analysis of severe-dose AHI and risk of T2DM using random-effects models. (b) Galbraith radial plot for assessment of publication bias among all included studies in the severe-dose subgroup meta-analysis. (c) Sensibility assessment of each included in the severe-dose subgroup meta-analysis. (d) Funnel plots for assessment of publication bias among all included studies in the severe-dose subgroup meta-analysis.

All the included studies researched the correlation between mild-dose AHI (5–15) and the risk of occurrence of T2DM. Three observational studies paid attention to the effect of moderate-to-severe-dose AHI (>15). Only two studies reported the association between moderate-dose AHI (15–29) and T2DM. Moreover, some research did not select 0 < AHI < 4.9 as a reference, which led to difficulty in merging these data. Besides, Appleton et al. [19] only performed the studies in the men cohort, and the age for the participants was significantly younger than other groups. This study scored the lowest in the NOS, which revealed the bias in further analysis. Due to the lack of moderate-dose studies and the inconsistent definition of moderate, we merged another moderate-to-severe subgroup with the moderate-to-severe subgroup in the traditional meta-analysis. The results of the mild different subgroups were OR = 1.23 (95% CI = 1.06–1.41, *P* = 0.002) (Figure 1(a)) for mild subgroup, OR = 1.35 (95% CI = 1.13–1.61, *P* < 0.001) for moderate (Figure 2(a)), OR = 2.14 (95% CI = 1.72–2.67, *P* < 0.001) for moderate-to-severe (Figure 3(a)), and OR = 2.19 (95% CI = 1.30–3.68, *P* < 0.001) for severe (Figure 4(a)).

### 3.4. Bias Examination and Heterogeneity

We applied Egger's and Begg's texts for the bias examination. The *P* < 0.05 means that there existed bias in the selection of the studies. The *P* value of Begg's or Egger's texts for all subgroups was more than 0.05. The results of the heterogeneity in the different subgroups were as follows: 0 (*P* = 0.47) for mild, 59% (*P* = 0.12) for moderate, 0 (*P* = 0.8) for moderate-to-severe, and 79% (*P* = 0.008) for severe. For further heterogeneity research, the funnel plot (Figures 1(d), 2(d), and 3(d)) and Galbraith radial plot (Figures 1(b), 2(b), and 3(b)) read that in each group except the severe-dose subgroup, all the studies were in the 95% confidence interval. These three studies were distributed around confidence interval boundaries for the severe-dose group, which led to a huge heterogeneity (Figures 4(b) and 4(d)).  Figure 4(c) reads that the lack of Kendzerska et al. [20] would greatly affect severe subgroup results in the meta-analysis estimations. The other meta-analysis estimations and the influence taken by each research were acceptable (Figures 1(c), 2(c), and 3(c)).

### 3.5. Dose-Response Meta-Analysis

**Figure 5:**
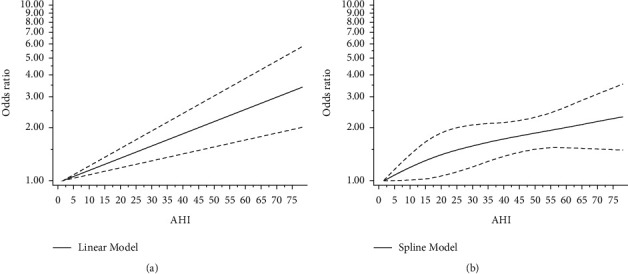
(a) Linear dose-response relationship between AHI and the risk of T2DM. The dashed line represents 95% CI. (b) Spline dose-response relationship between AHI and the risk of T2DM. The dashed line represents 95% CI.

There were statistically significant differences in the risk of T2DM between the mild-dose, moderate-to-severe-dose, and zero-dose groups of OSA. What is more, the linear model test result was not significant (*P* = 0.428), which means the existence of a linear model. We performed linear (Figure 5(a)) and spline models (Figure 5(b)). The linear analysis read that the risk of occurrence of T2DM increased by 1.62% for each event per hour increase in AHI (OR = 1.016, 95% CI = 1.009–1.023; *P* < 0.05). The spline analysis showed that the risk of occurrence of T2DM increased with increasing OSA severity.

## 4. Discussion

In the present study, we performed a traditional meta-analysis and a dose-response meta-analysis to thoroughly explore the correlation between OSA and the risk of T2DM. All the included primary studies concluded that OSA is a risk factor for the new occurrence of T2DM. However, not all the OSA dose subgroups were associated with T2DM. Appleton et al. [19] indicated that only the severe-dose AHI affected the incidence of T2DM (OR = 2.7, 95% CI = 1.3 to 5.4). Three articles reported that the mild-dose AHI was not related to the incidence of T2DM [19, 21, 22]. Moreover, the estimation of the effect of OSA on the risk of T2DM was not accurate. Marshall et al. [22] reported that the moderate-dose AHI affected the incidence of T2DM (OR = 8.62, 95% CI = 1.14 to 65.20), which was suspect for its limitation of samples scale. The results of Egger's or Begg's texts indicated there existed no bias in the selection of research works in the present study, which revealed the reliability of our study.

The risk for T2DM was associated with OSA severity across different OSA stages. Furthermore, the positive correlation between AHI and OR of T2DM indicated the possibility of a dose-response relationship.

### 4.1. Analysis of Heterogeneity

For the meta-analysis of two subgroups, the mild-dose subgroup and moderate-to-server-dose AHI analysis showed heterogeneity when merging the related research works. The mild-dose, moderate-dose, and moderate-to-severe-dose subgroups' heterogeneity showed no significant differences; the severe-dose subgroups were gross (*P* = 0.008, *I*^2^ = 79%). This suggested the result of the conventional meta-analysis was receivable, but accepting should be cautious. We speculated heterogeneity might be for the following reasons. First, there were different proportions of age and gender participants in the included studies. Male OSA has higher AHI compared to age-matched females [23]. Older age OSA has higher AHI than younger ones [24]. The prevalence is higher in males than in females [25]. Second, different studies adjusted different items in the research. For example, obesity is an essential confounder for the occurrence of T2DM. Higher weight is usually associated with higher AHI [26]. Reichmuth et al. [27] selected waist girth for the body habitus measures. However, others used body mass index (BMI). Different measuring methods and items between included studies might lead heterogeneity when merging the related research works. Finally, some studies in the included passages shared the different research types. Some studies were cross-sectional studies, while the left one belonged to cohort studies. The differences between OR and RR might count in a traditional meta-analysis.

### 4.2. Analysis of Dose-Response Meta-Analysis

#### 4.2.1. Comparison with Similar Studies

Our study provided strong evidence that OSA was related to the risk of T2DM occurrence and performed a prediction for the RR to different AHI doses with the linear and spline models. The results were consistent with previous meta-analyses [28–30]. In Qie et al. [28], the authors performed a similar dose-response meta-analysis of OSA and DM's relationship. However, we set a stricter criterion for the included research works and enlarged the number of included studies. Besides, due to the linear model test result, we performed both spline and linear models in the dose-response analysis, which provided more information than only a linear model. Wang et al. [29] and Tatti et al. [30] reported that OSA was closely related to T2DM but did not evaluate the RR of T2DM at different AHI doses.

#### 4.2.2. Possible Mechanisms

OSA is characterized by intractable hypoxemia, which leads to various pathologic conditions, including neural activation, systemic inflammation, oxidative stress loading, and hormone disorder. The changes in hormonal systems will add an influence on energy metabolism. Xu et al. [31] and Zhang et al. [32] have proved that sleep disorder will lead to insulin resistance by enhancing oxidative stress in vivo. Conversely, T2DM will disorganize the respiratory system during sleep time and aggravate OSA. These mechanisms reveal that OSA is correlated to the incidence of T2DM independent of other factors like age or obesity. This circulation accompanies each OSA patient for an extended period and will cause other health problems.

### 4.3. Limitations

There are a few impediments to our research. First, some studies' sample size was limited. Marshall et al. [22] only researched 303 samples. Fewer subjects might bring a low confidence level. This might be a potential reason for the heterogeneity. Second, when construing the linear and spline models, we needed as much data as possible. The limitation of the number of included studies brought errors for the regression. Third, the measurement of OSA was inconsistent. Finally, the different measurement machines might apply influence to the AHI.

## 5. Conclusion

Our research has proved that OSA is a risk factor for DM. Besides, for different severity, OSA shows a different OR. As a quantitative indicator of OSA, the AHI was positively related to the risk of DM, which indicates that doctors should pay more attention to the patients' breath events during sleep after ablation treatment. In this research, we set up a dose-response model for AHI and DM. With the help of the model we built, physicians will have more evidence to decide the intervention time for OSA patients to prevent DM.

In a word, our study concluded that the AHI is positively correlated to the risk of DM occurrence. OSA is associated with the occurrence of T2DM. Further studies are needed to assess whether OSA treatment would decrease T2DM risk and benefit the management of T2DM.

## Figures and Tables

**Table 1:** The criteria for inclusion and exclusion.

Criteria for inclusion	Criteria for exclusion
Patients with type II diabetes	All kinds of reviews, case reports, or fundamental research works
Research providing accurate risk ratio or odds ratio and 95% confidence intervals	No clear outcome or selected type I diabetes as case outcomes
All kinds of cross-section studies, cohort studies, or case-control studies	Without a control group
Studies are divided into subgroups by the AHI or RDI and the mean AHI (RDI) dose of each subgroup was clearly declared	Used other indicators except AHI/RDI
	No integrated risk ratio or odds ratio

**Table 2:** The baseline characters of the included studies.

Publication	Study location	Study type	Female count (%)	Age (years)	Follow-up period (years)	OSA categories	Adjustment for confounder	Definition of diabetes	Participants	Number of diabetic patients	Diabetes count (%)
Bakker 2015	USA	Cohort study	54.20%	Mean 68.5 ± 9.2 years	3	None: 0 ≤ AHI ≤ 4.9 events/hr. Mild: 5 ≤ AHI ≤ 14.9 events/hr. Moderate-to-severe AHI ≥ 15 events/hr.	Study site, age, gender, and ethnicity	Glucose ≥ 126 mg/dL and/or hypoglycemic medication use	2151	865	40.21

Reichmuth 2005	USA	Cohort study	43.63%	Mean 49.0 ± 8.3 years	4	None: 0 ≤ AHI ≤ 4.9 events/hr. Mild: 5 ≤ AHI ≤ 14.9 events/hr. Moderate-to-severe AHI ≥ 15 events/hr.	Sex, age, and waist girth and waist girth × sex interaction	FPG ≥ 7.0 mmol/L or report of physician-diagnosed diabetes	1387	58	4.18

Nagayoshi 2016	USA	Cohort study	43%	45–64 years mean 63.0	13	None: 0 ≤ AHI ≤ 5 events/hr. Mild: 5 ≤ AHI ≤ 15 events/hr. Moderate 16 ≤ AHI ≤ 29 events/hr. Severe AHI ≥ 30 events/hr.	Age, sex, and center	Report of physician-diagnosed diabetes or use of diabetes medication	1453	285	19.61

Marshall 2009	Australia	Cohort study	28.38%	Mean 53.2	4	None: 0 ≤ RDI ≤ 4.9 events/hr. Mild: 5 ≤ RDI ≤ 14.9 events/hr. Moderate-to-severe RDI ≥ 15 events/hr.	Age, gender, and waist circumference	FPG ≥ 7.0 mmol/L or report of a physician diagnosis or treatment of diabetes	303	29	9.57

Appleton 2015	Australia	Cohort study	0	Mean 60.5	4.7	None: 0 ≤ AHI ≤ 10 events/hr. Mild: 11 ≤ AHI ≤ 19 events/hr. Moderate 20 ≤ AHI ≤ 29 events/hr. Severe AHI ≥ 30 events/hr.	Age	FPG ≥ 7.0 mmol/L or HbA1c of ≥ 6.5% or self-reported diabetes diagnosis or treatment of diabetes	736	66	8.97

Botros 2009	USA	Cohort study	6.63%	Mean 61.5	5	Quartile #1 AHI < 8 events/hr. (reference group); quartile #2, 8 ≤ AHI ≤ 20; quartile #3, 21 ≤ AHI ≤ 45; quartile #4, AHI ≥ 46	Unadjusted	Diabetes was defined by a physician diagnosis during routine office visit and fasting blood glucose 126 mg/dL	544	61	11.21

Kendzerska 2014	Canada	Cohort study	38.00%	48(38–58)	5.6	None: 0 =< AHI =< 5 events/hr. Mild: 5 =< AHI =< 15 events/hr. Moderate 16 =< AHI =< 29 events/hr. Severe 30 =< AHI events/hr.	Sex, age, body mass index, history of smoking status, prior comorbidities (HTN, AMI, ADG categories) and income	a validated algorithm that identifies people with diabetes as those having at least one; hospitalization record or at least two physician services claims bearing a diagnosis of diabetes within a 2-year period	8678	1017	11.72

**Table 3:** The NOS of each included study.

Article	Selection	Comparability	Outcome	Total
Reichmuth 2005	^*∗∗∗∗*^	^*∗*^	^*∗∗*^	7
Bakker 2015	^*∗∗∗∗*^	^*∗∗*^	^*∗∗*^	7
Nagayoshi 2016	^*∗∗∗∗*^	^*∗*^	^*∗∗∗*^	8
Botros 2009	^*∗∗∗∗*^	^*∗∗*^	^*∗∗*^	8
Appleton 2015	^*∗∗∗∗*^	^*∗∗*^	^*∗∗*^	8
Kendzerska 2014	^*∗∗∗∗*^	^*∗∗*^	^*∗∗∗*^	9
Marshall 2009	^*∗∗∗∗*^	^*∗∗*^	^*∗∗∗*^	9

## Data Availability

All data are included in the article.

## Supplementary Materials

Supplementary Materials Seven articles were identified with our meta-analysis criteria in total (Supplementary Materials 1). The search strategy in PubMed is shown in Supplementary Materials 2.
