# Supplementary material for: Association between Obstructive Sleep Apnea and Type 2 Diabetes Mellitus: A Dose-Response Meta-Analysis
Source: Evid Based Complement Alternat Med. 2021 Sep 30;2021:1337118. doi: 10.1155/2021/1337118 (PMC8497107; doi:10.1155/2021/1337118)
Supplement: Supplementary Materials — Seven articles were identified with our meta-analysis criteria in total (Supplementary Materials 1). The search strategy in PubMed is shown in Supplementary Materials 2. [file 1337118.f1.zip › 1337118.f1/supplementary material 1.doc]

**PRISMA 2009 Flow Diagram**

**Screening**

**Included**

**Eligibility**

**Identification**

Records identified through database searching
(n =537)

Additional records identified through other sources
(n =1)

Records after duplicates removed
(n =489)

Records screened
(n =329)

Records excluded
(n =160)

Full-text articles assessed for eligibility
(n =39)

Full-text articles excluded, with reasons

not relevant (n = 290)

Studies included in qualitative synthesis
(n =13)

Studies included in quantitative synthesis (meta-analysis)
(n =7)
