# Supplementary material for: Association between Obstructive Sleep Apnea and Type 2 Diabetes Mellitus: A Dose-Response Meta-Analysis
Source: Evid Based Complement Alternat Med. 2021 Sep 30;2021:1337118. doi: 10.1155/2021/1337118 (PMC8497107; doi:10.1155/2021/1337118)
Supplement: Supplementary Materials — Seven articles were identified with our meta-analysis criteria in total (Supplementary Materials 1). The search strategy in PubMed is shown in Supplementary Materials 2. [file 1337118.f1.zip › 1337118.f1/supplementary material 2.doc]

(((((((((((((((((((((((((((((((((Diabetes Mellitus, Type 2[Title/Abstract]) OR (Diabetes Mellitus, Noninsulin-Dependent[Title/Abstract])) OR (Diabetes Mellitus, Ketosis-Resistant[Title/Abstract])) OR (Diabetes Mellitus, Ketosis Resistant[Title/Abstract])) OR (Ketosis-Resistant Diabetes Mellitus[Title/Abstract])) OR (Diabetes Mellitus, Non Insulin Dependent[Title/Abstract])) OR (Diabetes Mellitus, Non-Insulin-Dependent[Title/Abstract])) OR (Non-Insulin-Dependent Diabetes Mellitus[Title/Abstract])) OR (Diabetes Mellitus, Stable[Title/Abstract])) OR (Stable Diabetes Mellitus[Title/Abstract])) OR (Diabetes Mellitus, Type II[Title/Abstract])) OR (NIDDM[Title/Abstract])) OR (Diabetes Mellitus, Noninsulin Dependent[Title/Abstract])) OR (Diabetes Mellitus, Maturity-Onset[Title/Abstract])) OR (Diabetes Mellitus, Maturity Onset[Title/Abstract])) OR (Maturity-Onset Diabetes Mellitus[Title/Abstract])) OR (Maturity Onset Diabetes Mellitus[Title/Abstract])) OR (MODY[Title/Abstract])) OR (Diabetes Mellitus, Slow-Onset[Title/Abstract])) OR (Diabetes Mellitus, Slow Onset[Title/Abstract])) OR (Slow-Onset Diabetes Mellitus[Title/Abstract])) OR (Type 2 Diabetes Mellitus[Title/Abstract])) OR (Noninsulin-Dependent Diabetes Mellitus[Title/Abstract])) OR (Noninsulin Dependent Diabetes Mellitus[Title/Abstract])) OR (Maturity-Onset Diabetes[Title/Abstract])) OR (Diabetes, Maturity-Onset[Title/Abstract])) OR (Maturity Onset Diabetes[Title/Abstract])) OR (Type 2 Diabetes[Title/Abstract])) OR (Diabetes, Type 2[Title/Abstract])) OR (Diabetes Mellitus, Adult-Onset[Title/Abstract])) OR (Adult-Onset Diabetes Mellitus[Title/Abstract])) OR (Diabetes Mellitus, Adult Onset[Title/Abstract])) AND ((((((((((((((Sleep Apnea, Obstructive[Title/Abstract]) OR (Apneas, Obstructive Sleep[Title/Abstract])) OR (Obstructive Sleep Apneas[Title/Abstract])) OR (Sleep Apneas, Obstructive[Title/Abstract])) OR (Obstructive Sleep Apnea Syndrome[Title/Abstract])) OR (Obstructive Sleep Apnea[Title/Abstract])) OR (OSAHS[Title/Abstract])) OR (Syndrome, Sleep Apnea, Obstructive[Title/Abstract])) OR (Sleep Apnea Syndrome, Obstructive[Title/Abstract])) OR (Apnea, Obstructive Sleep[Title/Abstract])) OR (Sleep Apnea Hypopnea Syndrome[Title/Abstract])) OR (Syndrome, Obstructive Sleep Apnea[Title/Abstract])) OR (Upper Airway Resistance Sleep Apnea Syndrome[Title/Abstract])) OR (Syndrome, Upper Airway Resistance, Sleep Apnea[Title/Abstract]))) AND (risk*[Title/Abstract] OR risk*[MeSH:noexp] OR risk *[MeSH:noexp] OR cohort studies[MeSH Terms] OR group*[Text Word])
